# From Viscount Knutsford

**Published:** 1918-07-27

**Authors:** 

**Affiliations:** Chairman, London Hospital. London Hospital, Whitechapel, E. 1


### From Viscount Knutsford.

Sir,—Sir Henry Burdett has no authority to voice "the nursing profession." And to write on July 13 that my letter of the 12th "has caused universal regret throughout the nursing world" is to constitute himself a central exchange as well. Neither he nor his son-in-law, Colonel Maurice, has given any reason why the London Hospital should change the admittedly good training of its nurses. The results speak for themselves. The Matrons-in-Chief both in England and in France are both London Hospital trained. The War Office is employing 218 of our nurses, and others are waiting for orders. We have had to refuse 90 urgent applications for nurses during last week alone. Nothing more need be said; at any rate I will say no more.—Yours faithfully,

Knutsford, Chairman, London Hospital.

London Hospital, Whitechapel, E. 1, July 15.

